# Anti-Ige monoclonal antibody therapy for the treatment of patients with chronic rhinosinusitis: a multi-disciplinary practice review

**DOI:** 10.1186/1710-1492-10-S2-A24

**Published:** 2014-12-18

**Authors:** Shaun J Kilty, Andrea Lasso, Stephanie Santucci, William Yang

**Affiliations:** 1 The Department of Otolaryngology-Head and Neck Surgery, The Ottawa Hospital, Ottawa, ON, Canada; 2 Ottawa Hospital Research Insitute (OHRI), Ottawa, ON, Canada; 3 The University of Ottawa, Ottawa, ON, Canada; 4 Allergy and Asthma Research Centre, Ottawa, ON, Canada

## Background

Several treatment options have been described for chronic rhinosinusitis (CRS), yet many patients remain poorly responsive to medical and surgical therapy. Recently, anti-IgE monoclonal antibody has emerged as a potential therapy for CRS. However, to date evidence for its efficacy in this patient population is sparse. The purpose of this study is to evaluate the clinical effect of anti-IgE monoclonal antibody therapy for patients with recalcitrant CRS and asthma treated in a multi-disciplinary clinic.

## Methods

A review of the charts for the 194 patients on anti-IgE monoclonal antibody therapy was performed. 20 patients diagnosed with CRS with poorly controlled disease having failed surgical and/or medical therapy were identified. Data extraction targeted demographic details, asthma, environmental allergy and CRS specific disease related data. For data analysis, for nonparametric data the Mann-Whitney test was used and for binary data Fisher’s exact test was used.

## Results

Mean age of the cohort was 49 years (range 33-67); eleven patients were male. Mean IgE level was 331.14 IU/ml (57.54-1338.96 IU/ml). Mean treatment duration was 17 (3-71) months. The most common skin prick test positive environmental allergens were dust mite (100%) and cat (65%). 75% of patients had CRS with polyps. Six patients (30%) had AERD. The mean polyp score decreased from 1.8 to 1.0 (p=0.106). Patient olfaction improved in 11 patients (55%) with therapy. Two patients on chronic prednisone treatment were able to discontinue this treatment. None of the patients progressed to require surgical treatment.

## Conclusions

Anti-IgE monoclonal antibody therapy allowed for clinical CRS disease control in this cohort of patients with severe and recalcitrant CRS. A well-designed clinical trial is needed to further assess the efficacy and safety of this treatment in the CRS population.

